# Camouflaging, not sensory processing or autistic identity, predicts eating disorder symptoms in autistic adults

**DOI:** 10.1177/13623613241245749

**Published:** 2024-04-18

**Authors:** Siofra Bradley, Fhionna Moore, Fiona Duffy, Lili Clark, Tasha Suratwala, Pooky Knightsmith, Karri Gillespie-Smith

**Affiliations:** 1 The University of Edinburgh, UK; 2 NHS Tayside, UK; 3 NHS Lothian, UK; 4 NHS England, UK; 5 Creative Education, UK

**Keywords:** autism, Autistic identity, camouflaging, eating disorders, sensory processing

## Abstract

The objective of this study was to explore the role that Autistic identity, sensory processing and camouflaging behaviours have on eating disorder symptomology in Autistic adults. Previous research has focused on cognitive and sensory factors to explain the association between autism and eating disorders, but the roles of social identity and camouflaging are yet to be explored. Autistic participants (*N* = 180) were recruited from NHS settings and community groups. The participants completed online questionnaires measuring autistic identity, camouflaging behaviours, sensory processing, autistic traits and eating disorder symptoms. Multiple regression revealed that camouflaging significantly predicted eating disorder symptoms. Although sensory processing was related, it did not significantly *predict* eating disorder symptom severity. In addition, there was no significant relationship between autistic identity and eating disorder symptom severity. This study highlights the impact that camouflaging behaviours and sensory processing can have on eating disorder symptomatology in autism and may indicate important considerations for the treatment of eating disorders in Autistic people.

**Lay Abstract:**

This study aimed to explore the impact of Autistic identity (i.e. feeling like you belong to the Autistic community), sensory profiles (e.g. being over or under responsive to sensations) and camouflaging behaviours (i.e. masking) on eating disorder symptoms in Autistic adults. 180 Autistic people were recruited from the community and NHS. The Autistic people completed online questionnaires measuring Autistic identity, sensory profiles, camouflaging behaviours, autistic traits and eating disorder symptoms. The analysis showed that higher levels of camouflaging behaviour predicted higher levels of eating disorder symptoms. Sensory profiles were related to but did not predict eating disorder symptoms and there was no relationship between level of Autistic identity and eating disorder symptoms. This shows that camouflaging is the most important predictor of eating disorder symptoms in Autistic people, and warrants further exploration.

## Introduction

### Autism and eating disorders

Autism^1^ is a lifelong neurodevelopmental condition characterised by differences in communication, social functioning, patterns of behaviour and restricted interests (American Psychiatric Association (APA), 2013). A recent systematic review reported the global prevalence of autism to be around 1% of the population, with a male to female ratio of 4:2 (Zeidan et al., 2022). However, these figures must be interpreted with caution, given the issues associated with autism assessment cost, as well as the underdiagnosis of Autistic females (Adamson et al., 2020; Hull & Mandy, 2017; Milner et al., 2019).

Research has consistently shown that Autistic people have higher rates of other neurodivergent conditions and mental health difficulties than non-Autistic people, including eating disorders (EDs) (Lai et al., 2019). EDs include anorexia nervosa (AN), bulimia nervosa (BN), binge eating disorder (BED) and, more recently, avoidant/restrictive food intake disorder (ARFID; which is a feeding disorder; Carter Leno et al., 2022). NICE (2017) reports an overall estimate of 700,000 people in the United Kingdom with a diagnosis of EDs, 90% of whom are females. However, it is recognised that EDs in males are significantly underdiagnosed and only 1% of ED research is conducted with males (Lavender et al., 2017; Murray et al., 2017).

Despite limited prevalence research, reports show that Autistic individuals are twice as likely to experience EDs compared with their neurotypical counterparts (Sedgewick et al., 2021).

Systematic reviews have reported autism prevalence rates within clinical populations of AN and BN to range between 4.7% and 22.9% (Huke et al., 2013; Nickel et al., 2019). Positive relationships between autistic traits and ED symptoms have also been found in non-clinical populations (Barnett et al., 2021; Christensen et al., 2019).

Those who are Autistic and have EDs have been found to experience longer admissions in inpatient settings, with more severe presentations of EDs and poorer outcomes (Nielsen et al., 2015, 2022). Autistic people who have had ED treatment have reported their treatment experiences as being characterised by a lack of understanding and accommodations for autism, which has impacted their recovery and relationships with clinicians (Kinnaird et al., 2019). Due to a lack of research, there is no robust clinical guidance on treatment options for this population which may explain why ED clinicians have shared that they feel underconfident treating people with this presentation (Kinnaird et al., 2017).

As far as the authors are aware, to date, there has only been one clinical pathway designed for treating Autistic people with AN, taking into account sensory and cognitive profiles common to Autistic people. This is the PEACE (Pathway for Eating Disorders and Autism developed from Clinical Experience) Pathway (Tchanturia et al., 2021). This has showed extremely promising results, including reduced length of inpatient stay, increased clinician confidence in working with Autistic people with AN, and NHS savings equating to approximately £22,837 per patient and approximately £275,000 per year for the ED service as a whole (Tchanturia et al., 2021).

### Explanations for ED in autism

Up until now, both quantitative and qualitative research have focused on cognitive, social and sensory aspects of autism that may contribute towards the development and maintenance of EDs among Autistic people (Brede et al., 2020; Christensen et al., 2019; Kinnaird et al., 2019). Significant levels of cognitive factors associated with autism such as inflexible thinking, rigidity and theory of mind difficulties have been reported among ED populations, such as those with AN (Christensen et al., 2019; Kinnaird et al., 2019), but there is less evidence of this association in other EDs such as BED (Kelly et al., 2013; Manasse et al., 2015).

The directionality of this association remains a topic of debate. Seemingly, Autistic characteristics such as cognitive rigidity may be prevalent in those with EDs as a temporary result of starvation on the brain (Hiller & Pellicano, 2013). In contrast, some evidence has shown that in women who have recovered from EDs, high levels of autistic characteristics have still been found (Bentz et al., 2017; Nazar et al., 2018). Furthermore, there is growing evidence to suggest that autistic traits from childhood such as social communication difficulties may increase vulnerability for developing EDs (Westwood et al., 2018). Solmi et al.’s (2021) longitudinal research demonstrated that autistic traits in 7-year-olds predicted their disordered eating behaviour at age 14.

#### EDs and sensory processing

The relationship between sensory processing issues and common eating behaviours in autism has recently been highlighted by Nimbley and colleagues’ (2022) systematic review. Different sensory profiles such as hyper- and hyposensitivities have been found among Autistic people (Crane et al., 2009) and it has been proposed that these sensitivities can contribute to EDs. For example, food restriction may be a coping mechanism for distressing hyper-sensory experiences (Brand-Gothelf, 2016; Kinnaird et al., 2020), while hyposensitivity to internal sensations may result in difficulties interpreting hunger cues (Brede et al., 2020).

Brede et al.’s (2020) qualitative study with parents, healthcare workers and Autistic women with AN extracted themes of sensory overload, food-specific sensory sensitivities and internal and bodily sensations as contributing to Autistic women’s EDs. Treatment settings such as those with bright or loud inpatient wards and meal plans involving certain food textures, smells and tastes can make ED recovery particularly challenging (Kinnaird et al., 2019).

#### EDs and social identity

A theme highlighted by qualitative accounts and case studies of Autistic people with EDs is the role that social identity played in the development and maintenance of their ED (Brede et al., 2020; Dandil et al., 2019). It has been proposed that the development of an ED could be related to identity issues that arise from being Autistic. Autistic people are part of a minority and are often a stigmatised group both before and after diagnosis (Botha & Frost, 2020; MacLeod et al., 2013). Accounts of Autistic people with EDs have suggested that AN may provide an identity or group to belong to (i.e. people who have AN) or be a form of coping due to not fitting in (Brede et al., 2020). This taken together highlights the need to investigate the role that Autistic identity plays on ED symptomology.

Tajfel et al.’s (1979) Social Identity Theory (SIT) framework suggests that if people are a member of a stigmatised group, they engage in attempts to regain a positive identity. They do this by either promoting the in-group status (joining support groups or Autism rights organisations) or dissociating from this group and identifying with a more ‘socially accepted’ group. Many Autistic people are rightly proud of their Autistic identity and appreciate the strengths that are associated with being Autistic (R. Cooper et al., 2021). Positive social identification with Autism has been associated with mental health benefits such as better self-esteem and lower levels of anxiety and depression (Cage et al., 2018; K. Cooper et al., 2017; R. Cooper et al., 2021). However, this has not been explored as a potential protective factor against EDs specifically in Autistic people.

#### EDs and camouflaging

‘Camouflaging’ or ‘masking’ is an attempt to dissociate from autism by engaging in behaviours that appear non-Autistic such as maintaining eye contact and rehearsing facial expressions (Bargiela et al., 2016; Beck et al., 2020). Autistic people have reported that camouflaging has been utilised as a response to autism stigma, in an attempt to mask their difficulties, fit in to a non-autistic world and identify with social groups (Bargiela et al., 2016; Hull & Mandy, 2017; Perry et al., 2022). This camouflaging, however, leads to increased levels of mental and physical exhaustion (Bargiela et al., 2016; Beck et al., 2020), with a recent review reporting that higher levels of camouflaging relate to poorer mental health outcomes (Cook et al., 2021). However, the relationship between camouflaging and ED outcomes has not yet been explored.

Similar to ED prevalence, camouflaging appears to be more prevalent among Autistic females than males (Lai et al., 2017) which may evoke speculation that EDs and camouflaging could be interlinked. This has been corroborated by Autistic females’ accounts in ED inpatient settings where they often report to engage in camouflaging behaviours (e.g. copying others and adopting their anorexic values) as a way of fitting in to a neurotypical world (Brede et al., 2020). Further evidence has been provided by caregivers who highlight that AN may emerge from a reduced ability to cope in more complex social situations as Autistic young people get older (Adamson et al., 2020). Given the implications of the associations between camouflaging and ED development and maintenance for Autistic people, it is surprising that this relationship has not been investigated.

### Study aims

The aim of the current study is to explore the role of Autistic identity, camouflaging behaviours and sensory processing in ED symptomology severity. The purpose of this is to add greater understanding towards the development and maintenance of EDs in autism, providing information to help inform treatment pathways for Autistic people with EDs and prevent poor outcomes.

### Study predictions

It is predicted that a strong Autistic identity will predict lower levels of ED symptomology. It is also predicted that higher levels of camouflaging will predict higher levels of ED symptomology, and finally high levels of sensory processing issues will predict high levels of ED symptomology.

## Methods

### Recruitment

This study received ethical approval from the NHS West Midlands Solihull Research Ethics Committee and NHS Tayside to act as a Participant Identification Centre.

Participants completed questionnaires online via the Qualtrics Survey platform from May 2022 to October 2022. Online recruitment was chosen for this study due to possible disruptions that COVID-19 imposed and because previous research highlighted that Autistic people often prefer online platforms as it reduces social demands (K. Cooper et al., 2017; Hendrickx, 2015). There were no incentives or reimbursements offered for participation in this study.

Clinicians within NHS Tayside Eating Disorder service (TEDS) and Autism service (TAACT) informed eligible patients about the study. Social Media ads containing the link to the online Qualtrics platform were placed on social media platforms, as well as university and charity webpages.

### Participants

Participants were included in this study if they were able to read English, were over the age of 18 and had either a clinical or self-diagnosis of Autism. Including people who self-identify as Autistic as this is a more inclusive practice endorsed by the Autistic community. In addition, research shows that those who self-identify as Autistic are accurate at recognising autistic traits in themselves and score similarly in autism scales, employment struggles, stigma and quality of life measures (McDonald, 2020; Overton et al., 2023). Participants were not excluded if they had intellectual disabilities and were asked to indicate this on the demographic section of the survey. No one indicated that they had an intellectual disability.

A priori power calculations using a G*Power calculator (Faul et al., 2009) was used to compute an adequate sample size for a regression analysis with five predictor variables. The calculation was based on a statistical power of 0.80 (Cohen, 1988), an alpha level of 0.5 (Green, 1991) with a medium effect size of *F*^2^ = 0.15 which calculated that a sample of 92 participants would be required for this study. The current study initially recruited *N* = 265 participants, of which some had to be removed due to missing or incomplete data (see ‘Data analyses’ section for more information), leaving the final sample as *N* = 180.

Participants remained anonymous throughout; however, demographic information was gathered. The age range of participants was 18–70 with an average age of 38 (*SD* = 13.6) years. Most participants had a clinical diagnosis (80.6%, *N* = 145) rather than a self-diagnosis (19.4%, *N* = 35) of Autism. Participants mostly consisted of females (64.4%, *N* = 116), followed by males (20%, *N* = 36), transgender (10.6%, *N* = 19) and those who preferred not to say (5%, *N* = 9). Most participants were from a White ethnic background (87.2%, *N* *=* 157), followed by Mixed or Multiple ethnicities (6.7%, *N* = 12), Black or African American or Caribbean (2.8%, *N* = 5), Asian (2.8%, *N* = 5) and Other (0.6%, *N* = 1). Participants were asked if they have ever received a diagnosis of an ED in their lifetime. The majority of participants (63.9%, *N* = 115) responded ‘Yes’ while a smaller amount reported ‘No’ (36.1%, *N* = 65). Table 1 presents the types of diagnosis that people reported as well as the frequency of other mental health diagnoses that people currently had.

**Table 1.** Participant demographic information.

Characteristics	*N* (%)
Age in years (*n* = 8 missing)	38 (*SD*: 13.6)
Gender	
Female	116 (64.4)
Male	36 (20)
Transgender	19 (10.6)
Prefer not to say	9 (5)
Ethnicity	
White	157 (87.2)
Mixed or multiple ethnicities	12 (6.7)
Black or African American or Caribbean	5 (2.8)
Asian	5 (2.8)
Other	1 (0.6)
Autism diagnosis	
Clinical diagnosis	145 (80.6)
Self-diagnosis	35 (19.4)
Eating disorder diagnosis	
No	115 (63.9)
Yes	65 (36.1)
Frequency of eating disorder types	
Multiple	23 (35.3)
Anorexia nervosa	22 (33.8)
Bulimia nervosa	6 (9.2)
Binge disorder	5 (7.7)
Other	4 (6.2)
OSFED (other specified feeding and eating disorder)	2 (3.1)
ARFID (avoidant/restrictive food intake disorder)	2 (3.1)
Did not say	1 (1.5)
Other mental health diagnosis	
No	64 (36)
Yes	116 (64.4)

This table demonstrates the mean age and standard deviation of participants. It also demonstrates the number, total percentage and amount of missing data for each characteristic.

### Measures

The study contained five questionnaires to gather information on the variables of autistic traits, sensory processing, camouflaging and Autistic identity, with another questionnaire measuring the outcome variable – ED symptomology. It took participants approximately 30 min to complete the study.

#### Autistic traits

The Autism Spectrum Quotient (AQ-10; Allison et al., 2012) is a shortened 10-item version of the 50-item Autism Quotient scale used to indicate levels of autistic traits, with higher scores indicating more autism characteristics. The maximum score for the AQ-10 is 10. The AQ-10 was used in this study for descriptive purposes to indicate participant autistic trait levels, and was not used as a screening instrument to verify clinical status. Critically, it has previously demonstrated sensitivity of 0.88, specificity of 0.91 and positive predictive value of 0.85 and internal consistency of (α > 0.85) among male and female samples (Allison et al., 2012). The AQ-10 displayed moderate reliability in this study (*a* = 0.63).

#### Camouflaging

Levels of camouflaging were measured using the Camouflaging Autistic Traits Questionnaire (CAT-Q; Hull et al., 2019). This questionnaire contains 25 statements which participants respond to using a Likert-type scale to indicate how much they agree or disagree with each statement. The total scores range from 25 to 175, with higher scores indicating higher camouflaging behaviours. The CAT-Q has previously demonstrated good test–retest reliability (*r* = 0.77) and high internal consistency for camouflaging (α = 0.94) (Hull et al., 2019). In this study, the CAT-Q displayed robust internal consistency (*a* = 0.82).

#### Autistic identity

The Social Identification Scale (SIS; Leach et al., 2008) is a 14-item scale which has shown good reliability (α = 0.87) across a range of identities. It can be adapted to measure Autistic identity by putting the phrase ‘Autistic person/people’ at the start of each question (K. Cooper et al., 2017) and has shown good internal consistency (α = 0.91). Participants rate on a 7-point Likert-type scale how much they agree or disagree with each statement. It has two dimensions: self-investment which is made up of solidarity (α = 0.82), satisfaction (α = 0.84) and centrality (α = 0.78), and self-definition which is comprised of self-stereotyping (α = 0.86) and in-group homogeneity (α = 0.66). The SIS demonstrated strong internal consistency in this study (*a* = 0.90).

#### Sensory processing

The Sensory Processing Quotient (SPQ; 10 items; Greenberg et al., 2018) was used to measure participant’s levels of sensory sensitivity across the five senses. The SPQ-10 showed good internal consistency from this study (α = 0.85). Participants respond to 10 statements using a Likert-type scale to indicate how strongly they agree or disagree with each statement. The maximum score that can be obtained is 30, with higher scores indicating higher levels of sensory sensitivities.

#### Eating disorder symptom severity

The Eating Disorder Examination Questionnaire Short (EDE-QS; Gideon et al., 2016) was used to measure ED behaviours and attitudes (from here on referred to as ED symptomology) by asking participants to consider the preceding 7 days when responding to 12 items on a 4-point response scale. It is a shortened version of the Eating Disorder Examination Questionnaire (EDE-Q) 28-item commonly used in research concerned with the overlap between autism and EDs (Kerr-Gaffney et al., 2020; Westwood et al., 2017). A maximum score of 36 can be obtained with higher scores indicating more disordered eating symptomology and scores of 15 or above indicating potential ED cases in clinical settings (Prnjak et al., 2020).

The EDE-QS has been highly correlated with the EDE-Q (*r* = 0.91 for people without EDs; *r* = 0.82 for people with EDs) and displayed high internal consistency in this study and in previous studies (α = 0.91) (Gideon et al., 2016). It has shown good levels of specificity (0.85), sensitivity (0.83) and positive predictive value (0.37) (Prnjak et al., 2020).

### Data analyses

The data were exported from Qualtrics to a password-protected excel file which was only accessible to members of the research team. The data were analysed using IBM SPSS V22. Data cleaning occurred to remove erroneous data before analyses occurred. Data sets were removed if they did not consent to take part (*N* *=* 32), were less than 85% completed (*N* *=* 48), completed in less than 260 s as this may have implied automated responses (*N* *=* 3), and completed as part of a preview or mock responses (*N* *=* 2). There were no missing data for any of the predictor variables and only 1.7% missing data for the outcome variable (EDE-QS), due to this low proportion no further action was required at the pre-analysis stage.

The data were explored to determine whether it met assumptions for parametric tests. The variance inflation factor (VIF) for each variable was below 2, indicating that the data met the assumption of collinearity. The assumption of normality of residuals yielded conflicting results. Assumptions of normality of residuals were checked through viewing histogram and Q-Q plots, and scatterplots of the standardised residuals and predicted values indicated that the assumptions of homoscedasticity and linearity were also met.

Bivariate correlations were conducted to establish which predictor variables showed a significant relationship with the outcome variable of ED symptomology and only significant results were taken forward and entered into the regression model. A stepwise multiple regression was then performed to test the study predictions. The autistic traits predictor was entered into the model first as this was the control variable, followed by the predictor variables based on the strength of their relationship with the outcome variable of eating disorder symptom severity. The predictor variable of sensory processing demonstrated the weakest relationship and was entered first, followed by camouflaging. The regression coefficients produced from the multiple regression were explored to examine the unique variance that each predictor variable contributed to the ED symptomology outcome.

### Community involvement

This study was co-produced by members from the Autistic community with lived experience following participatory research guidelines (Fletcher-Watson et al., 2019). A peer Autistic researcher with lived experience of EDs helped with the initial conceptualisation of the project, ensuring relevance to the Autistic community. Two Autistic mentors also helped select appropriate measures, looked over participant documents and were involved in the writing (and co-authorship) of this article.

## Results

### Means and standard deviations

Table 2 presents the means and standard deviations for each of the independent variables (see Table 2).

**Table 2.** Mean score and standard deviation of each variable.

Variable	*M*	*SD*
ED symptoms	6.86	4.86
AT	7.87	1.90
SP	19.74	6.37
CB	115.48	17.99
AI	66.32	13.91

ED symptoms: eating disorder symptoms; AT: Autistic traits; SP: sensory processing; CB: camouflaging behaviours; AI: Autistic identity.

### Correlations

Table 3 presents correlations between the variables that were significant with the outcome variable of disordered eating symptomology (see Table 3).

**Table 3.** Correlations between variables and ED symptoms.

	AT	AI	CB	SP	ED symptoms
Autistic traits (AT)	–	0.331** (<0.001)	0.264** (<0.001)	0.230** (<0.013)	0.190* (<0.013)
Autism identity (AI)		–	0.353** (<0.001)	0.184* (<0.013)	–0.021 (0.761)
Camouflaging behaviours (CB)			–	0.326** (<0.001)	0.302** (<0.001)
Sensory processing (SP)				–	0.225** (<0.013)

ED: eating disorder.

* Correlation is significant to 0.013 (following Bonferroni correction; two-tailed).

** Correlation is significant to 0.001.

Pearson’s correlation tests were performed with a Bonferroni-adjusted alpha level of 0.013 (0.05/4) to explore the presence and strength of relationships between the variables. Weak to moderate positive and significant correlations were found between ED symptomology and camouflaging behaviours (*r* = 0.30, *p* < 0.001), sensory processing (*r* = 0.23, *p* *<* 0.013), and autistic traits (*r* *=* 0.19, *p* < 0.013). A negative correlation was found between ED symptomology and Autistic identity; however, this was not significant (*r* = –0.02, *p* *=* 0.761).

Although it was not related to the study’s predictions, interesting relationships were found between variables as shown in Table 3. Camouflaging and Autistic identity showed a significant positive relationship (*r* = 0.35, *p* < 0.001) Camouflaging behaviours showed significant positive relationships with autistic traits (*r* *=* 0.26, *p* < 0.001), and sensory processing (*r* = 0.33, *p* < 0.001). Sensory processing displayed significant positive relationships with autistic traits (*r* = 0.23, *p* *<* 0.013).

There was no significant relationship found between ED symptomology and Autistic identity. Therefore, this variable was not included as a predictor variable for ED symptomology in further regression analyses.

### Stepwise multiple regression

A stepwise multiple regression was conducted to further analyse the significant relationships found between ED symptomology and the predictor variables of sensory processing and camouflaging. Only three variables were entered into the following regression model which meant the Bonferroni-adjusted alpha applied to the regression analyses was 0.017 (0.5/3). As autistic traits were significant at the *p* < 0.013 level, they were treated as a control variable and entered into the model first. Model 1, with autistic traits as the only predictor, explained 3.1% of the variance and was significant (*F*(1, 178) = 6.64, *p* < 0.017). Model 2, in which Sensory Processing was added, explained significantly more variance (*R*^2^ change = 0.035, *F*(1, 177) = 6.61, *p* < 0.017). The model significantly predicts 6% of the variance in ED symptomology (adjusted *R*^2^ = 0.060) (*F*(2, 177) = 6.73, *p* < 0.017). Model 3, in which camouflaging was added, explained more variance, and this increase was significant (*R*^2^ change = 0.047, *F*(1, 176) = 9.39, *p* < 0.017). Model 3 significantly predicted 10.3% of the variance in ED symptomology outcomes (adjusted *R*^2^ = 0.103) and was significant (*F*(3, 176 = 7.83, *p* < 0.001).

The regression coefficients showed the unique variance that each predictor variable contributed to ED symptomology outcomes. Model 3 shows that camouflaging (β *=* 0.24, *p* *<* 0.016) contributed unique variance to ED symptomology outcomes. Autistic traits (β *=* 0.10, *p* = 0.186) and sensory processing (β *=* 0.13, *p* *=* 0.099) did not contribute unique significant variance to ED symptomology outcomes, see Table 4.

**Table 4.** Stepwise multiple regression model.

	Variable	*B*	β	*p*	*df*	*F*	*p*	*R*^2^Adj
(EDE-QS)								
Model 1					1, 178	6.636	0.001	0.031
	AT	0.486	0.190	0.011				
Model 2					2, 177	6.728	0.001	0.071
	AT	0.373	0.145	0.052				
	SP	0.146	0.191	0.011				
Model 3					3, 176	7.829	0.003	0.118
	AT	0.253	0.099	0.186				
	SP	0.096	0.126	0.099				
	CB	0.063	0.235	0.003				

AT: Autistic traits; SP: sensory processing; CB: camouflaging behaviours.

## Discussion

### Findings

This study explored the role that Autistic identity, sensory processing and camouflaging may have on ED symptomology in Autistic people. The predictions were partly supported and the results show that high levels of camouflaging behaviours uniquely predicted ED symptomology in Autistic people. Although sensory processing was related, it was not found to significantly predict ED symptomology. In addition, there was no evidence to support the prediction that strong Autistic identity predicted lower levels of ED symptoms.

### Camouflaging and ED symptoms

This is the first study that quantitatively demonstrates the role of camouflaging on ED symptoms in Autistic individuals. These findings corroborate Autistic people and their parent’s accounts that EDs are related to camouflaging behaviours and that they are developed as a way of coping with social demands in a neurotypical world (Adamson et al., 2020; Brede et al., 2020). These findings uniquely add to existing research that have reported high levels of camouflaging behaviours are associated with poor mental health outcomes (Bargiela et al., 2016; Beck et al., 2020) by extending this further to ED symptoms.

### Autistic identity and ED symptomology

For the first time, the relationship between Autistic identity and ED symptomology was explored and it is somewhat surprising that a significant relationship was not found between these concepts. Previous research which also measured Autistic identity reported significant negative relationships between Autistic identity and mental health outcomes, indicating that Autistic identity is a protective factor against poor mental health (Cage et al., 2018; K. Cooper et al., 2017; R. Cooper et al., 2021). This study suggests that Autistic identity as a protective factor may not extend to ED symptoms or may indicate that current scales that measure ED symptomology do not adequately capture ED symptoms in Autistic groups (e.g. rules around eating may be viewed as being part of being Autistic and thus might not be captured by a measure focusing on traditional ED symptom).

### Sensory processing and ED symptomology

Sensory sensitivities and ED symptomology showed a positive relationship which supports previous findings about the role of sensory issues and eating behaviours in Autistic groups (Nimbley et al., 2022). It is worth noting, however, that although sensory sensitivity significantly explained variance in ED symptomology; when added to the regression model, it did not explain a large amount. It also did not significantly explain unique variance, indicating that although these concepts are related, sensory sensitivities do not appear to strongly predict ED symptomatology in autism.

The relationship between sensory sensitivities and AN has been highlighted (Brede et al., 2020); however, this has not been consistently corroborated (Kinnaird et al., 2020). In addition, there is even less research exploring the relationship between sensory sensitivities and other EDs, such as BED, and the few studies that have explored this have found no relationship (Kelly et al., 2013). Participants in this study reported either not having an ED or reported a range of Eds, including binge disorder, BN, OSFED (other specified feeding and eating disorder), and also ARFID which is a Feeding Disorder (Hay et al., 2017). This may explain the weaker predictive value of sensory processing and may suggest that sensory issues are more prevalent in some EDs than others. Furthermore, this finding may also have been again caused by the EDE-QS measure being developed based on more typical ED presentations and may not adequately capture the range or differing ED presentations that are reported to co-occur with autism (Huke et al., 2013).

### Strengths and limitations

It is essential to investigate the various factors that contribute to all types of EDs and disordered eating behaviours among Autistic people. This study has added to the research base by exploring sensory processing, Autistic identity and camouflaging factors for the first time in terms of ED symptomology. Despite this strength, there are limitations associated with the study that must be noted. This includes the limited availability of existing measures to capture Autistic identity. The SIS was not originally developed for measuring Autistic identity; however, it was the only appropriate tool available that had previously been adapted and used to measure Autistic identity (K. Cooper et al., 2017).

In addition, the EDE-QS which was used to capture ED symptomology contained items that aimed to measure constructs such as weight and shape concerns (Gideon et al., 2016). Therefore, it may not have been an appropriate measure for some of the eating symptomology experienced by the current Autistic participants, particularly if this ED symptomology is not driven by weight or shape concerns such as those who have ARFID (Hay et al., 2017). Recent research has improved understanding about the potential social and sensory motivations in Autistic people who have AN (Nimbley et al., 2023) and similar work needs to be carried out for other eating and/or feeding disorders.

The cross-sectional design and regression analyses method used in this study limits the conclusions that can be made in terms of the directionality of the relationships between camouflaging and sensory processing with ED symptomology. There may be other confounding variables that were not accounted for in this study that explain the relationships, for example, thwarted belongingness or cognitive factors.

This design of this study took participant burden into consideration and chose to use shortened versions of measures such as the SPQ-10 and the EDE-QS which displayed good reliability and validity (Prnjak et al., 2020). Despite a potential strength of this study being the online recruitment method (which allowed participants from various geographical locations and demographics to take part), this may have also been a limitation potentially causing a biased sample, with those most likely to have a strong Autistic identity to take part. Related to this a large demographic majority of this study were White (87.2%) and female (64.4%) which means that caution should be taken when applying the findings of these results to all Autistic people (those who have high support needs, trans and non-binary, children, etc.).

In addition to the potential bias in the current sample, no methods were used to verify clinical diagnoses of autism and self-identified Autistic people were also included. Participant recruitment routes were not recorded (due to the nature of the survey) and it is not known how many participants were recruited from the NHS or community. Also, despite the AQ-10 being used to indicate levels of autistic traits, it was not used as a screening tool as research has suggested its poor reliability in this regard (Taylor et al., 2020). Therefore, although this study endorsed a more inclusive research practice, caution must be warranted when interpreting the results.

### Future research

Future research needs to begin looking at whether these factors or others (i.e. Autism acceptance or Autism collective self-esteem) predict or protect against ED symptomology and the different types of ED diagnoses. This study explored Autistic identity; however, future studies should also explore the potential negative impact that ED identity may have on Autistic people with a diagnosis of ED. As this study has been the first to demonstrate a relationship between camouflaging and ED in an Autistic population with and without diagnoses of ED, future studies could explore this relationship among Autistic people, including those with a clinical diagnosis of ED. Longitudinal research is required to determine the direction of the significant relationships found within this study. In addition to this, more underrepresented Autistic groups need to be included in ED research to better understand the underpinning mechanisms that contribute to ED diagnoses; these groups include those who have non-White ethnicity, non-cis gender and those who have high support needs.

### Recommendations

This study has highlighted the need for the development of reliable and validated measures to assess Autistic identity given importance of this concept in terms of SIT and autism being an historically stigmatised group. This study also emphasises the need for increased efforts and funding to reduce the stigma of autism and create a more neurodiversity-affirming environment. Reducing the stigma is likely to reduce the need for Autistic people to engage in camouflaging behaviours, leading to poor mental health outcomes. This is particularly important as this study adds to this literature by showing that camouflaging predicts ED symptomology. It may be worth clinicians working with Autistic people to consider screening for camouflaging behaviours to inform their assessments and formulation of mental health difficulties such as EDs. Finally, it is important to further develop and validate scales that measure ED symptomology such as the EDE-QS; to ensure that it measures the range of EDs with varying ED presentation in Autistic groups.

## Conclusion

This study has uniquely added to understanding the relationship between autism and ED symptomatology by highlighting the impact that camouflaging behaviours and sensory processing have on ED symptomology in Autistic people. This emphasises the need to increase awareness of camouflaging behaviours and their impact on EDs symptoms in Autistic groups. Although Autistic identity was not identified as a significant factor in this study, more research should consider its measurement and the role it may play in different types of EDs. More quantitative and qualitative research should be conducted to extend the findings of this study and provide a more in-depth understanding of the roles of sensory processing and camouflaging in Autistic groups who have EDs.

## Notes

1. The words *Autism* and *Autistic* have been capitalised throughout this article when referring to matters related to identity or Autistic community as this reflects the neurodivergent perspective that Autism is an identity rather than a medical condition.
